# Clinical value of preferred endoscopic ultrasound-guided antegrade surgery in the treatment of extrahepatic bile duct malignant obstruction

**DOI:** 10.1016/j.clinsp.2022.100017

**Published:** 2022-03-13

**Authors:** Xuan Zhao, Lihong Shi, Jinchen Wang, Siming Guo, Sumin Zhu

**Affiliations:** Department of Gastroenterology, The Second Affiliated Hospital of Xuzhou Medical University, Xuzhou, China

**Keywords:** Malignant obstruction of extrahepatic bile duct, Endoscopic ultrasound, Smooth, Endoscopic retrograde pancreatic bile angiography, Drainage

## Abstract

**Objectives:**

To explore the clinical value of preferred ultrasound endoscopic guided biliary drainage in patients with extrahepatic biliary obstruction with intrahepatic biliary ectasis.

**Methods:**

A total of 58 patients with malignant obstruction and intrahepatic bile duct expansion, including 32 males, 26 females and median age 65 (58‒81) were selected. A prospective randomized controlled study was randomized into EUS-AG and ERCP-BD, with 28 patients in EUS-AG and 30 in ERCP-BD. The efficacy of the two treatments, operation success rate, operation time, the incidence of complications, hospitalization days, cost, unimpeded stent duration, and survival time were compared.

**Results:**

1) The surgical success rate in group EUS-AG was 100%, and in group, ERCP-BD was 96.67%. There was no statistical difference in surgical success rate in the two groups (p>0.05). 2) Average operating time in EUS-AG was (23.69±11.57) min, and in ERCP-BD was (36.75±17.69) min. The difference between the two groups has statistical significance (p<0.05). 3) The clinical symptoms of successful patients were significantly relieved. Compared with the preoperative procedure, the differences in group levels had statistical significance (p<0.05); TBIL, ALP, WBC and CRP levels, no statistical significance difference in groups (p>0.05).

**Conclusion:**

EUS-AG operation has short time, low incidence of complications, safe, effective, and can be used as the preferred treatment plan for patients with extrahepatic biliary duct malignant obstruction associated with intrahepatic biliary duct expansion; EUS-AG operation has more unique clinical advantages for patients with altered gastrointestinal anatomy or upper gastrointestinal obstruction.

## Highlights

• The aim of the research is to explore the clinical value of preferred ultrasound endoscopic guided biliary drainage in patients with extrahepatic biliary obstruction with intrahepatic biliary ectasis.
• A prospective randomized controlled study was randomized into EUS-AG and ERCP-BD, with 28 patients in EUS-AG and 30 in ERCP-BD.
• The authors found the EUS-AG operation can be done in a short time, low incidence of complications, is safe, effective, and can be used as the preferred treatment plan for patients with extrahepatic biliary duct malignant obstruction.

## Introduction

Endoscopic retrograde cholangiopancreatography guided biliary drainage (ERCP-guided Biliary Drainage, ERCP-BD) is the first choice for biliary drainage in patients with malignant biliary obstruction. In patients with endoscopic access to the duodenal papilla, the current success rate of ERCP intubation is 95% or above. However, due to tumor invasion, postoperative anatomical structure changes, gastroduodenal obstruction, and other factors, there are still cases where the endoscope cannot reach or enter the duodenal papilla, resulting in the failure of ERCP.^1^ Percutaneous Transhepatic Cholangiodrainage (PTCD) is a traditional palliative interventional therapy for patients with malignant biliary obstruction after ERCP failure. However, PTCD often occurs some complications, such as bile loss, electrolyte disturbance, intestinal dysfunction, acute cholangitis, and skin infection. Moreover, carrying the drainage bag outside the body for a long time also seriously affects the quality of life of patients. Endoscopic Ultrasound-Guided Biliary Drainage (EUS-BD) has gradually become an effective alternative therapy after the failure of ERCP.^2^ Studies have indicated that the clinical success rate of EUS-BD can reach 92%∼100%.^3^, ^4^, ^5^ In addition, a number of reports have suggested that EUS-BD can not only be used as an alternative for the failure of ERCP-BD, but also as a potential first-line treatment for malignant biliary obstruction.^6^, ^7^, ^8^ This research aims to compare the therapeutic value of EUS-Antegrade (EUS-AG) in EUS-BD and ERCP-BD in patients with malignant extrahepatic bile duct obstruction and intrahepatic bile duct dilation, which is reported as follows.

## Materials and methods

### Research object

A total of 58 patients with malignant extrahepatic bile duct obstruction and intrahepatic bile duct dilatation were hospitalized in the Second Affiliated Hospital of Xuzhou Medical University and the Second Affiliated Hospital of Nanjing Medical University from January 2017 to January 2020 were collected. There were 32 males and 26 females with a median age of 65 (58‒81) years in this project. Clinical diagnosis showed 30 cases of cholangiocarcinoma, 13 cases of pancreatic cancer, 9 cases of gastric cancer metastasis, 4 cases of gallbladder cancer involvement, and 2 cases of colon cancer metastasis. This study was approved by the Ethics Committee of The Second Affiliated Hospital of Xuzhou Medical University ([2017]021201), this study was registered in the Chinese Clinical Trial Registry (ChiCTR) under number ChiCTR2100047026, and all patients signed informed consent.

Inclusion criteria: 1) Patients with malignant extrahepatic bile duct obstruction with intrahepatic bile duct dilatation have been confirmed using Imaging or pathological examination; 2) Patients who can't be performed radical surgical resection due to the advanced stage of malignant tumor or associated disease; 3) Patients have been agreed to sign the informed consent.

Exclusion criteria: 1) Patients who refuse to participate in the study; 2) Patients who have severe coagulation dysfunction or abnormal platelets; 3) Patients who have cirrhosis, severe esophageal stenosis, and severe varices of esophagogastric fundus; 4) Patients who accompany severe diabetes, refractory hypertension, serious insufficiency of vital organs; 5) Patients who cannot tolerate endoscopic surgery.

Surgeon criteria:^9^ 1) Doctors who have 4‒5 years of experience in endoscopic ultrasound and ERCP surgery; 2) Doctors who perform ERCP intubation, and the success rate is ≥95%.

### Equipment and materials

Endoscopic ultrasonography (GF-UCT260, Olympus); Electronic duodenoscope and accessories (TGF260V, Olympus); Nipple incision knife (ST0725, LeoMed); High-frequency generator (300D, ERBE); 0.035 zebra thread (JiuHong); 19G puncture needle (COOK); Dilating bougie (SBDC-6/7/8.5 Fr, COOK); Metal biliary stent (BONA).

### Therapy scheme and grouping

Preoperative preparation: Complete the routine preoperative examination of electrocardiogram, blood routine examination, liver function, hepatitis B immunity, virus, and coagulation function; perform imaging examinations such as color ultrasound, CT, or MRI to identify the lesion sites and evaluate the condition of illness. Antiplatelet aggregation and anticoagulant drugs were stopped 1-week before surgery. Intravenous broad-spectrum antibiotics were used to prevent infection preoperative 1-day. Patients with a clear diagnosis after admission and meeting the inclusion criteria were divided into different groups randomly.

**Figure 1:**
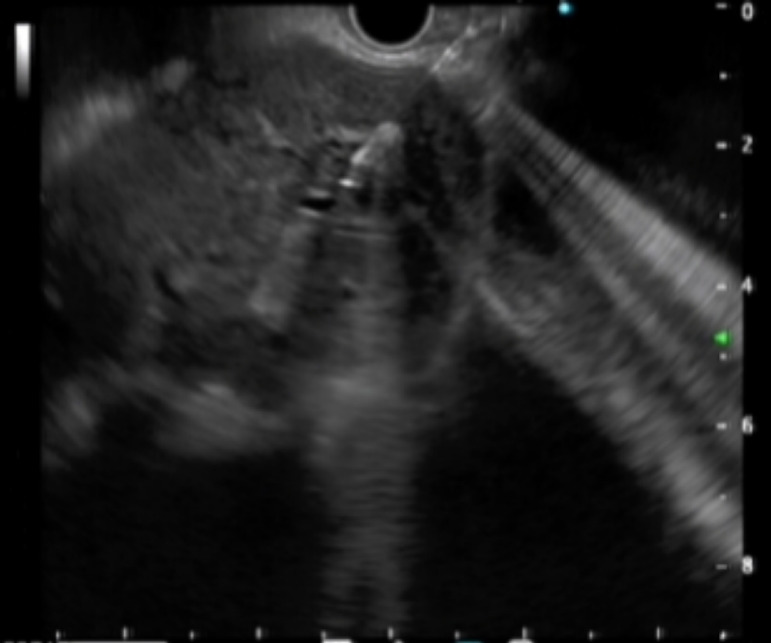
Ultrasound endoscopically guided intrahepatic bile duct puncture.

**Figure 2:**
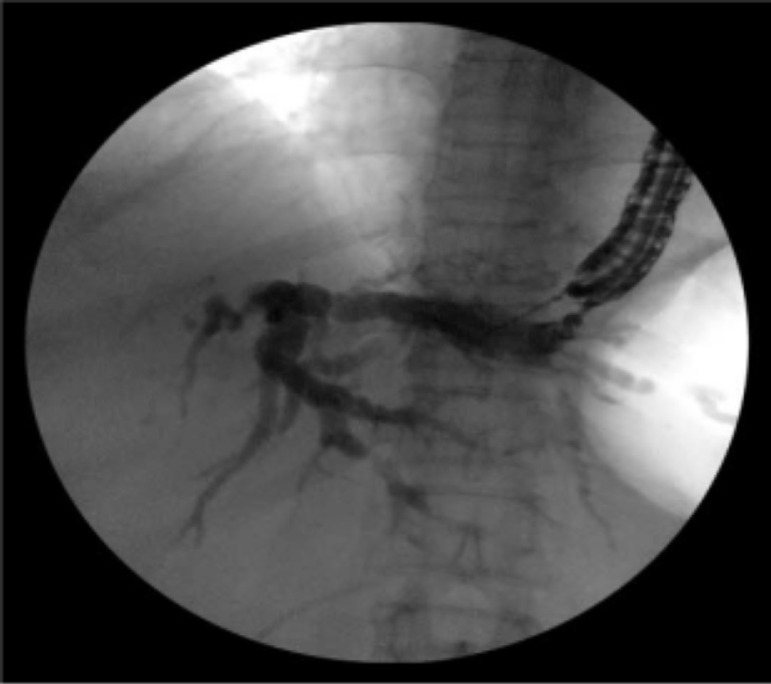
Location confirmed by cholangiography.

**Figure 3:**
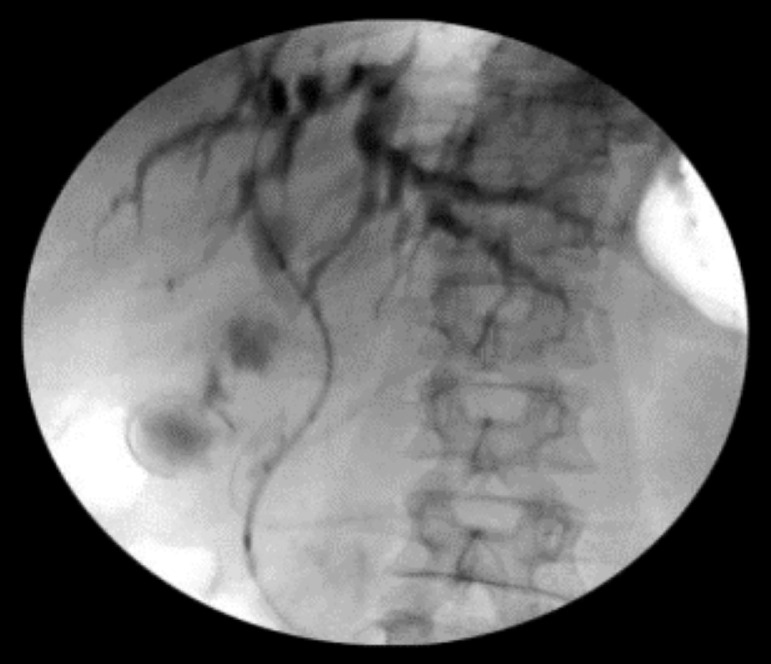
Guide wire placement and anterograde passage through the bile duct stricture.

**Figure 4:**
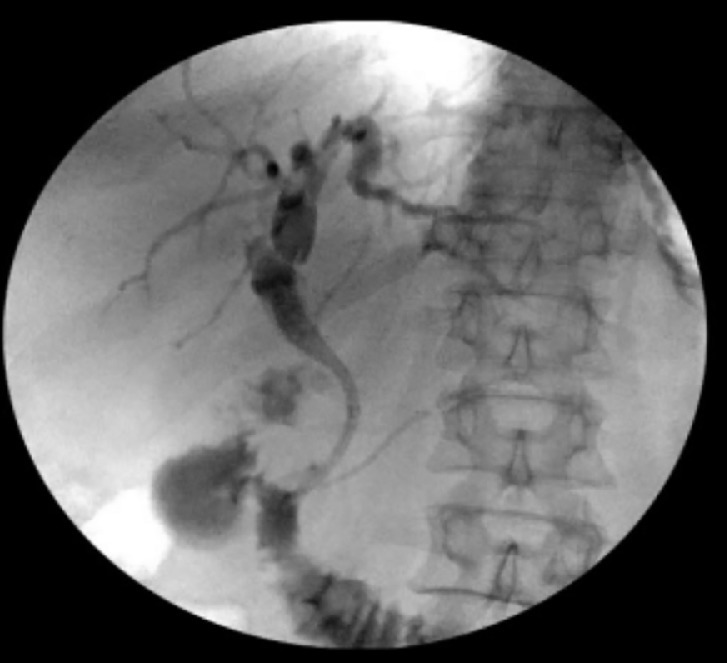
Stent placement and Cholangiography.

Intraoperative operating: EUS-AG group: ultrasound endoscopy was placed in the stomach to find the dilated intrahepatic bile ducts. Three segments of intrahepatic bile ducts with a dilated diameter greater than 5-mm were preferably selected, then adjusted the better puncture angle (upper left and lower right puncture). A 19G puncture needle was used to perform intrahepatic bile duct puncture under the guidance of endoscopic ultrasound, then extract bile, and a contrast agent was injected into the biliary tract to confirm the location. A 0.035 inch of wire was inserted along the needle tract, and the needle tract was expanded by using a probe according to the situation. ERCP-BD group: The patient was in a left decubitus position and entered the mirror to the descending part of the duodenum to find the nipple. Selective bile duct intubation was performed and guided by zebra guide wire via duodenal papilla. Cholangiography (ioversol) was used to determine the location, length, and extent of bile duct obstruction. The wire was inserted after the intubation was successful, then the wire crossed the stenosis and entered the bile duct/left and right intrahepatic bile ducts. Place the stent along the guidewire and confirm the stent expansion and smooth drainage (Fig. 1‒4).

Postoperative management: Patients were fasting, and electrocardio was monitored for 24‒48h after operation; Use proton pump inhibitor to suppress acid and protect stomach; Blood routine examination, blood amylase, liver function, and other related indicators were performed again at 24h; Closely monitor vital signs of patients.

### Observational index

Follow-up was conducted by outpatient or telephone, and the follow-up period was 12 months.

1) Operation success rate and operation time.
2) Clinical effects: TBIL, ALP, WBC, CRP, and blood amylase were reexamined before surgery and postoperative 3, 7, 14 days, and 1-month. The clinical symptoms were also evaluated. Clinical success refers to the decrease of bilirubin and the improvement or remission of clinical symptoms such as fever and abdominal pain.
3) Postoperative complications: They include hemorrhage, perforation, infection, cholangitis, biliary peritonitis, stent displacement, blockage, biliary fistula, hyperamylasemia, pancreatitis, and so on.
4) Stent-free period and median survival time of patients.
5) Hospitalization cost and hospital stays.
6) Median survival time.

### Statistical analysis

Statistical analysis was conducted with SPSS 16.0 (SPSS Inc. Chicago, IL). The data were normally distributed and shown as (Χ±s). Data that are not normally distributed are represented by the median. Comparison between the two groups was analyzed using unpaired Student's *t*-tests. The counting data were tested by χ2 test or Fisher's exact test; Kaplan-Meier method was used to calculate the biliary stent unimpeded time and median survival time; p<0.05 was considered statistically significant.

## Results

### General data

There were no significant differences in gender, age, Total Bilirubin (TBIL), Alkaline Phosphatase (ALP), White Blood Cell count (WBC) between 2 groups, and the differences were not statistically significant (p > 0.05), as shown in Table 1.

**Table 1:** Comparison of general data between the two groups.

**Group**	**Cases (man/women)**	**Median age (years)**	**TBIL (μmoL/L)**	**ALP (U/L)**	**WBC (109/L)**
EUS-AG	15/13	63(60‒81)	336.15±158.27	865.37±327.59	8.59±4.67
ERCP-BD	17/13	67(58‒79)	325.57±152.69	849.69±309.32	8.35±4.39
P	0.813	0.729	0.527	0.413	0.571

Note: EUS-AG, Endoscopic Ultrasound-Guided Antegrade Surgery; ERCP-BD, Endoscopic Retrograde Cholangio-Pancreatography guided Biliary Drainage; TBIL, Total Bilirubin; ALP, Alkaline Phosphatase; WBC, White Blood Cell.

### Operation success rate and average operation time

The surgical success rate was 100% (28/28) in the EUS-AG group and 96.67% (29/30) in the ERCP-BD group. In the ERCP-BD group, one patient had no success in ERCP intubation due to metastatic infiltration of the duodenal papilla, but EUS-AG succeeded instead. There was no statistically significant difference between the groups (p > 0.05).

The average operation time was (23.69 ± 11.57) min in the EUS-AG group and (36.75±17.69) min in the ERCP-BD group. The average operation time in the EUS-AG group was significantly shorter than that in the ERCP-BD group. There was a statistically significant difference between the groups (p < 0.05).

### Clinical efficacy

The postoperative jaundice of patients in the two groups gradually subsided, and the symptoms of fever and abdominal pain were gradually relieved. The TBIL level pre-operation, at postoperative 3, 7, 14 days, and 1-month in the EUS-AG group were (336.15 ± 158.27), (285.37 ± 119.59), (239.17 ± 113.59), (112.59 ± 92.57), (52.65 ± 27.87) μmol/L, respectively. While in the ERCP-BD group, TBIL level were (325.57 ± 152.69), (286.35 ± 108.79), (232.73 ± 106.57), (115.76 ± 96.59), (59.35 ± 30.27) μmol/L, respectively. Compared with preoperative surgery, there were statistically significant differences in the level of decline between the two groups (p < 0.05). The levels of TBIL, ALP, WBC, CRP, and the levels of decline in the same period between the groups were not statistically significant (p > 0.05), as shown in Table 2.

**Table 2:** The levels of related laboratory indicators before and after treatment in the two groups of patients (Χ ± s).

**Group**	**Time**	**TBIL (μmoL/L)**	**ALP (U/L)**	**WBC (109/L)**	**CRP (mg/L)**
EUS-AG (n=28)	Pre-operation	336.15±158.27	865.37±327.59	8.59 ±4.67	53.93±35.75
	Postoperative 3d	285.37±119.59^a^	765.59±285.37^a^	11.85±5.79^a^	72.35±36.51^a^
	Postoperative 7d	239.17±113.59^a^	587.38±229.75^a^	9.65±3.95	60.75±30.57
	Postoperative 14d	112.59±92.57^a,b^	428.69±119.69^a,b^	7.82±3.56^b^	32.95±19.52^b^
	Postoperative 1-month	52.65±27.87^a,b^	136.57±42.79^a,b^	7.58±3.35^b^	20.59±13.75^b^
ERCP-BD (n=30)	Pre-operation	325.57±152.69	849.69±309.32	8.35±4.39	51.51±35.75
	Postoperative 3d	286.35±108.79^a^	757.93±299.65^a^	12.15±5.39^a^	78.59±35.79^a^
	Postoperative 7d	232.73±106.57^a^	602.35±236.59^a^	9.73±4.15	65.71±33.65
	Postoperative 14d	115.76±96.59^a,b^	443.53±150.79^a,b^	8.16±3.97^b^	35.35±20.91^b^
	Postoperative 1-month	59.35±30.27^a,b^	141.93±47.65^a,b^	7.69±3.55^b^	22.69±15.95^b^

Note: EUS-AG. Endoscopic Ultrasound-Guided Antegrade Surgery; ERCP-BD, Endoscopic Retrograde Cholangio-Pancreatography guided Biliary Drainage; TBIL, Total Bilirubin; ALP, Alkaline Phosphatase; WBC, White Blood Cell; CRP, C-Reactive Protein.

Compared with pre-operation in this group: ^a^ p<0.05 compared with postoperative 3-days in this group;

bp < 0.05.

### Complications

The total postoperative complication rate in the EUS-AG group was 3.57% (1/28), and 1 case of biliary bleeding (melanorrhea, improved after symptomatic hemostasis treatment). The total postoperative complication rate in the ERCP-BD group was 26.67% (8/30), 3 cases of hyperamylaseemia (after the operation, the blood amylase was increased, the patient had no obvious abdominal pain, and CT showed no obvious abnormalities. The blood amylase was dynamically reexamined and gradually improved). 1 case of biliary hemorrhage (black stool, decreased hemoglobin, improved after symptomatic hemostatic treatment). One case of cholangitis (clinical manifestations of abdominal pain and fever, improved after antibiotic treatment). One case of mild pancreatitis (clinical manifestations of abdominal pain, combined with CT and blood amylase examination to confirm the diagnosis, fasting, somatostatin inhibition of pancreatic juice secretion, application of antibiotics, and nutritional support treatment improved after treatment). One case of duodenal perforation (It improved after fasting, gastrointestinal decompression, and antibiotics). One case of stent blockage (upper abdominal pain, fever, and jaundice repeated symptoms, examination showed that the stent was blocked, reoperation was performed, and a stent was implanted in the metal stent cavity After getting better). There were no serious complications related to surgery in both groups. The total postoperative complication rate in the EUS-AG group was lower than that in the ERCP-BD group, and the difference between the groups was statistically significant (p < 0.05).

### Hospitalization time and expenses

The average hospital stay in the EUS-AG group was (5.15 ± 3.57) days and (6.89 ± 3.91) days in the ERCP-BD group, respectively. There was no significant difference between the groups (p > 0.05). The average hospitalization cost in the EUS-AG group was (3432.97 ± 827.52) USD and (3461.32 ± 869.68) USD in the ERCP-BD group. There was no statistically significant difference between the groups (p > 0.05).

### Median unimpeded time of the stent and survival time

Kaplan-Meier method was used to calculate the median patency time of the stent, which was 252 days in the EUS-AG group and 241 days in the ERCP-BD group. There was no significant difference between the groups (p > 0.05). The median survival time was 152 days in the EUS-AG group and 147 days in the ERCP-BD group, and there was no statistically significant difference between the groups (p > 0.05).

## Discussion

Currently, ERCP-BD has become the preferred therapy for malignant bile duct obstruction. However, even experienced endoscopists still have a 5% to 10% failure rate of intubation.^10^ EUS-BD is an effective remedial treatment after the failure of ERCP-BD. Its effectiveness and safety have been widely recognized by endoscopy experts at home and abroad.^8^^,^^11^ With the development of EUS-BD becoming more mature, more and more randomized controlled trials^7^^,^^12^ and Meta-analysis^6^^,^^13^ report that EUS-BD can be used as a first-line treatment for malignant bile duct obstruction. According to the drainage method, EUS-BD is mainly divided into anterograde drainage (EUS-AG), transmural drainage, and docking drainage (EUS-RV). At present, there is no unified recommendation on the specific surgical route and surgical method, which mainly depends on the etiology, the site of obstruction, whether the anatomical structure is being changed, whether the endoscope is reaching the duodenal papilla, and other factors. Among them, EUS-AG was first reported by Fujita et al.^14^ in 2008. Intrahepatic Bile Drainage (IHBD) was used to achieve the dual benefits of prolonging survival and improving the quality of life. It conforms to the characteristics of physiological and anatomical structure but has not been fully recognized. In this research, the authors compared EUS-AG and ERCP-BD biliary drainage as the first-line treatment options in a prospective plan for patients with malignant extrahepatic bile duct obstruction with intrahepatic bile duct dilatation, and the authors evaluated the clinical value of EUS-AG as the first-choice treatment for patients with malignant extrahepatic bile duct obstruction.

This research shows that compared with ERCP-BD, EUS-AG has a shorter operation time, a lower complication rate, and a definite curative effect. It has unique advantages in clinical application for patients with malignant extrahepatic bile duct obstruction and intrahepatic bile duct dilatation, especially for patients with gastrointestinal anatomical changes or upper gastrointestinal obstruction, which is consistent with the study of Iwashita et al.^15^ EUS-AG ultrasound endoscopy realizes real-time guidance, accurate positioning, precise treatment, real-time feedback and safety, and efficiency through intraoperative real-time, visualization, and radiation-free navigation corrects the deviation of intraoperative positioning and preoperative imaging examination results and performs abnormal anatomy The structure is also accurately positioned, which significantly improves the success rate of one-needle puncture, reduces the incidence of complications, and shortens the operation time, which is consistent with the study of Kawakubo et al.^8^ ERCP-BD requires an endoscopic retrograde pass through the nipple. In the treatment of patients with gastrointestinal anatomical structure changes or upper gastrointestinal obstruction, it is not only technically more difficult, but also often accompanied by a relatively higher complication rate; Skinner et al.^16^ have reported that the success rate of ERCP treatment for patients with anatomical changes due to surgery is only about 48%‒70%. However, with the increase in surgical patients, disease screening, and medical imaging technology development, the proportion of patients with abnormal anatomical structures has gradually increased. Studies have shown that EUS-AG has a clinical success rate of 92%‒100% in the treatment of patients with changes in anatomical structure.^4^^,^^5^^,^^17^ and the complication rate has not increased,^17^ with high effectiveness and safety. Compared with other surgical methods of EUS-BD, EUS-AG has an exclusive approach to patients with anatomical structure changes or duodenoscopes that are difficult to reach the nipple. IHBD can be used exclusively through the residual stomach or small intestine to the left lobe of the liver after the operation. Didenal anastomosis (EUS-CDS) and EUS-RV could not establish access. Although there are punctures and needle tract expansion during EUS-AG operation, it is necessary to be alert to the occurrence of postoperative biliary fistula. The surrounding liver parenchyma can fill the temporary fistula after IHBD route without the permanent fistula. Compared with the Extrahepatic Bile Drainage (EHBD) route, the risk of the biliary fistula is lower.^18^, ^19^, ^20^ In this study, there were no complications of biliary fistula in the EUS-AG group, which is consistent with studies such as Iwashita.^15^

## Conclusion

In summary, compared with ERCP-BD, EUS-AG has a shorter operation time, a lower complication rate, safer and more effective. It can be used as the first choice for patients with malignant extrahepatic bile duct obstruction with intrahepatic bile duct dilatation. For patients with gastrointestinal anatomical structure changes or upper gastrointestinal obstruction, it has more unique advantages and clinical application value. Since the present study is limited by sample size, the research results still need to be further verified by the multi-center and large-sample prospective randomized controlled studies.

## Authors' contributions

Conception and Design: Xuan Zhao, Sumin Zhu.

Acquisition of Data: Xuan Zhao, Lihong Shi, Jinchen Wang, Siming Guo, Sumin Zhu.

Analysis and Interpretation of Data: Xuan Zhao, Lihong Shi, Jinchen Wang, Siming Guo, Sumin Zhu.

Drafting the Article: Sumin Zhu.

Revising Article for Intellectual Content: Xuan Zhao, Lihong Shi, Jinchen Wang, Siming Guo, Sumin Zhu.

Final Approval of the Completed Article: Xuan Zhao, Lihong Shi, Jinchen Wang, Siming Guo, Sumin Zhu.

## Conflicts of interest

The authors declare no conflicts of interest.
